# 388. Does This Patient Have *C. difficile* Infection? A Systematic Review and Meta-Analysis

**DOI:** 10.1093/ofid/ofac492.466

**Published:** 2022-12-15

**Authors:** Fizza Manzoor, Saba Manzoor, Ruxandra Pinto, Kevin Brown, Bradley J Langford, Nick Daneman

**Affiliations:** University of Toronto, Toronto, Ontario, Canada; University of Toronto, Toronto, Ontario, Canada; University of Toronto, Toronto, Ontario, Canada; University of Toronto, Toronto, Ontario, Canada; Public Health Ontario, Toronto, Ontario, Canada; Sunnybrook Health Sciences Centre, University of Toronto, Toronto, Ontario, Canada

## Abstract

**Background:**

The clinical features of *Clostridioides difficile* infection overlap with many conditions. Accurate and early diagnosis of *C. difficile* is essential for reducing morbidity and mortality. We performed a systematic review to evaluate the diagnostic utility of clinical findings associated with *C. difficile*.

**Methods:**

We included all studies that reported clinical features of *C. difficile*, a valid reference standard test for confirming diagnosis of *C. difficile*, and a comparison among patients with a positive and negative test result. The MEDLINE, EMBASE, CINAHL, and Cochrane databases were searched up to September 2021. Meta-analyses using univariate and bivariate methods were used to determine estimates of sensitivity, specificity, and likelihood ratios (LRs).

**Figure:**
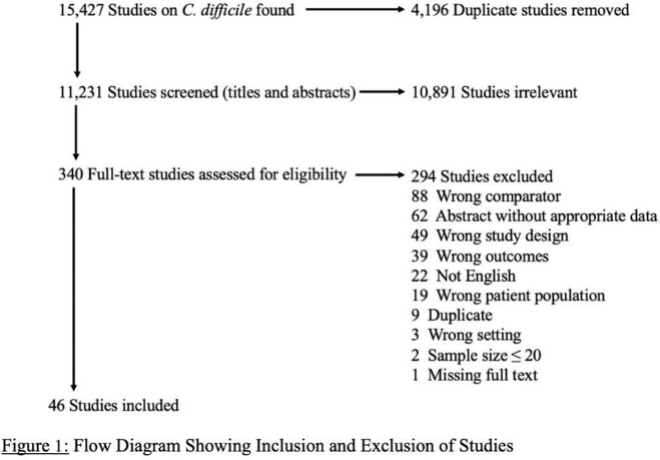

**Results:**

A total of 11,231 articles were screened and 46 were included for final analysis, enabling evaluation of 67 features for their diagnostic utility for *C. difficile* (10 clinical examination findings, 4 laboratory tests, 10 radiographic findings, prior exposure to 14 antibiotic types, and 29 clinical risk factors). Of the ten features identified on clinical examination, none were associated with increased likelihood of *C. difficile* infection. Features that increased likelihood of *C. difficile*infection were stool leukocytes (LR 5.31, 95% CI 3.29-8.56), hospital admission in prior three months (LR 2.39, 95% CI 1.68-3.30), leukocytosis (LR 1.50, 95% CI 1.24-1.75), and low serum albumin (LR 1.43, 95% CI 1.06-1.97). Some clinical co-morbidities increased likelihood of *C. difficile* including congestive heart failure (LR 3.01, 95% CI 2.26-3.80) and end-stage renal disease (LR 3.85, 95% CI 1.73-7.57). Several radiographic findings also strongly increased the likelihood of *C. difficile* infection like pericolonic stranding (LR 10.72, 95% CI 9.59-11.84) and ascites (LR 2.91, 95% CI 1.76-4.80).

**Figure:**
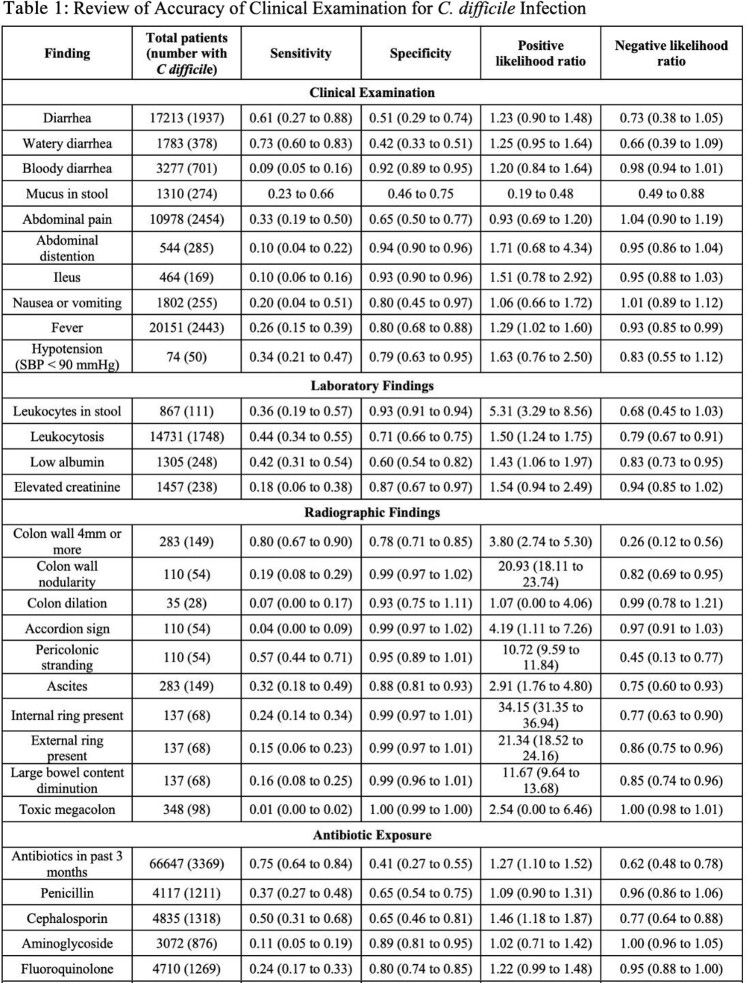

**Figure:**
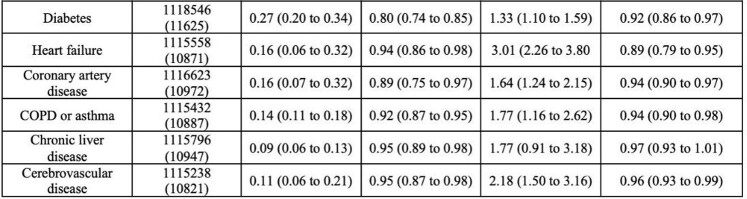

**Conclusion:**

There is limited utility of bedside clinical examination alone in detecting or ruling out *C. difficile* infection. Accurate diagnosis of *C. difficile* infection requires a combination of thoughtful clinical assessment and interpretation of test results. However, microbiologic testing is needed for confirmation in all suspected cases.

**Disclosures:**

**All Authors**: No reported disclosures.

